# Barriers and facilitators to recruitment and enrollment of HIV-infected individuals with opioid use disorder in a clinical trial

**DOI:** 10.1186/s12913-019-4721-x

**Published:** 2019-11-21

**Authors:** Kim A. Hoffman, Robin Baker, Lynn E. Kunkel, Elizabeth Needham Waddell, Paula J. Lum, Dennis McCarty, P. Todd Korthuis

**Affiliations:** 1 Oregon Health and Science University- Portland State University, School of Public Health, 3181 SW Sam Jackson Park Rd., CB669, Portland, OR 97239-3088 USA; 2 Department of Medicine, University of California-San Francisco, San Francisco, CA USA; 3 Department of Medicine, Section of Addiction Medicine, Oregon Health and Science University, Portland, OR USA

## Abstract

**Background:**

The CTN-0067 CHOICES trial tests implementation of extended-release naltrexone (XR-NTX) versus treatment-as-usual (TAU) for opioid use disorders (OUD) in HIV clinics to improve HIV viral suppression. The study team investigated recruitment strategies to elucidate the barriers and facilitators to recruitment and enrollment in the study.

**Main text:**

Methods: Semi-structured, in-depth, digitally recorded interviews were completed with study recruitment-related staff and medical providers (*n* = 26) from six participating HIV clinics in the fall of 2018. Interviews probed 1) factors that might prevent prospective participants from engaging in study recruitment and enrollment procedures and 2) strategies used by study staff that encourage eligible patient participation. Interviews were transcribed and thematically analyzed using a content analysis approach. Results: All respondents reported that barriers to recruitment and enrollment included challenging patient social and structural factors (e.g., homelessness or living environments with high substance use, criminal justice involvement), difficulty locating patients with unsuppressed HIV viral load and OUD within the HIV clinic, time-consuming study enrollment processes, and stigma around HIV and OUD which inhibited treatment seeking. Some respondents observed that distrust of research and researchers impeded recruitment activities in the community. A specific medication-related barrier was patient fear of opioid abstinence required prior to XR-NTX induction. Facilitators of recruitment included use of trusted peer outreach/recruitment workers in the community, hospitalizations that offered windows of opportunities for screening and XR-NTX induction, providing participant transportation, and partnerships with harm reduction organizations for referrals.

**Conclusions:**

Though study personnel encountered barriers to recruitment in the CHOICES study, persons with untreated HIV and OUD can be enrolled in multisite clinical trials by using enhanced recruitment strategies that extend outside of the HIV clinic. Employing peer outreach workers and collaborating with syringe service programs may be especially helpful in facilitating recruitment and merit inclusion in similar study protocols.

## Background

Substance use disorders are common in individuals with HIV [1–5] and untreated substance use disorders (SUD) are associated with increased HIV risk behaviors [6–8], decreased receipt of antiretroviral therapy (ART) ([9], 2000 [7, 10];), decreased ART adherence [7, 11–13], decreased HIV viral suppression [14, 15], greater HIV-related symptoms [16, 17], and higher hospitalization rates [18, 19]. Other adverse outcomes include decreased health-related quality of life [20], greater HIV-related symptoms [16], higher hospitalization rates [18], and greater HIV disease progression and death [14]. Treatment of SUD can increase engagement in HIV care and enhance health outcomes [14, 21].

### Opioid antagonist therapy for individuals with HIV and opioid use disorder

Naltrexone (NTX), a full mu-opioid antagonist, has FDA approval for treatment of opioid use disorder but is used infrequently because it requires daily dosing. A systematic review and meta-analysis of randomized studies found that oral naltrexone was not superior to placebo for the treatment of OUD. An extended-release formulation of naltrexone (XR-NTX) lasts 28 days and eliminates the need for daily dosing. XR-NTX was associated with improved adherence and retention in treatment for alcohol dependence [22] but has not been well tested in people living with HIV (PLWH). A recent study, however, found that it is more difficult to induct patients onto XR-NTX than buprenorphine [23], likely due to the negative-opioid urine sample required before XR-NTX induction. A 50-patient pilot study demonstrated the feasibility of XR-NTX for the treatment of opioid and alcohol use disorders in HIV primary care at two HIV clinics. Mean days of opioid use in the past 30 days decreased in both the treatment as usual (17.3 to 4.1 days) and the XR-NTX group (20.3 to 7.7 days) and HIV suppression improved from 67 to 80% for XR-NTX and 58 to 75% for treatment as usual [24].

### CTN-0067 CHOICES study

The National Drug Abuse Treatment Clinical Trials Network “Comparing Treatments for HIV-Infected Opioid Users in an Integrated Care Effectiveness Study (CHOICES) Scale-up study” (CTN-0067) is a randomized trial of HIV clinic-based XR-NTX versus TAU (other medications for opioid use disorder) for treatment of opioid use disorder that began recruiting participants in March 2018 (ClinicalTrials.gov: NCT03275350). Clinic staff at six HIV clinics in five states completed training in the research study protocol. The primary outcome is HIV viral suppression at 24 weeks. Participants must have a moderate or severe OUD and an elevated HIV viral RNA level of ≥200 copies/ml to be eligible. Study staff utilized a variety of traditional methods for study recruitment, including approaching prospective participants during clinic visits and the use of flyers. Preliminary results show that study recruitment and enrollment from HIV clinics have been more challenging than anticipated at the time the study was proposed. In recent years, the development of potent new ART agents (i.e., integrase inhibitors) facilitate rapid HIV viral suppression when PLWH engage in care. As such, the study eligibility criteria are being applied to a harder-to-reach population that is either undiagnosed or not engaged in HIV care. In response, sites expanded and experimented with different strategies in order to facilitate recruitment, including the use of outreach workers to engage with people in the community and recruit potential participants.

A qualitative investigation explored staff perspectives on the barriers and facilitators associated with study recruitment and enrollment of this hard-to-reach population during the trial’s early implementation phase. Interviews explored a) influences that inhibited eligible individuals from engaging in study recruitment and enrollment procedures and b) strategies used to encourage study participation.

## Main text

### Methods

Using an iterative group process, research questions and an interview guide were developed with the goal of better understanding clinic staff experiences during the study’s early implementation stages and challenges related to recruitment. We used a multiple-case, exploratory methodology (Mills, 2010). Two investigators conducted in-depth interviews via in-person and telephone interviews with participating staff (*n* = 26) from September to November 2018. Of the 26 research staff interviewed, 18 were women, 15 identified as white, non-Hispanic, and 15 had a Master’s degree or higher. The majority were study coordinators (*n* = 8), study clinicians (*n* = 7), or research assistants (*n* = 5). Clinical experience in the fields of either HIV or addiction medicine ranged from 1 to 31 years, and experience with medications for the treatment of opioid use disorder (MOUD) ranged from 1 to 24 years (Table 1). Interviews averaged 30 min in length. All interviewees were informed about confidentiality, freedom to participate and the right to withdraw from the study at any point. The Advarra Institutional Review Board reviewed and approved the study [25].

**Table 1:** Respondent Characteristics

VARIABLE	PARTICIPANTS (*n* = 26)
Age	
20–29	3
30–39	11
40–49	6
50–59	3
60–69	3
Gender	
Women	19
Men	6
Transgender	1
Education^a^	
Some college	1
Technical/associate degree	2
Bachelor	2
Master	8
PhD	5
MD	10
Study role	
Site PI	4
Study clinician	7
Study coordinator	8
Research assistant	5
Outreach worker	1
QA monitor	1
Years at clinic	
1–3	8
4–6	5
7–9	2
10–12	3
13–16	5
17–19	0
20+	3
Years experience with HIV or SUD	
1–3	4
4–6	6
7–9	2
10–12	2
13–16	6
17–19	2
20+	4
Years experience with MOUD	
1–3	15
4–6	4
7–9	1
10–12	3
13–16	2
17–19	0
20+	1

^a^Numbers will equal more than 26 as some participants reported more than 1 degree

*MOUD* Medications for opioid use disorder treatment (e.g. naltrexone, buprenorphine, and methadone)

Qualitative interviews were recorded and transcribed. ATLAS.ti 8.0 qualitative software facilitated coding, organization, and retrieval of text for analysis. Thematic codes were developed inductively as the transcripts were reviewed, allowing the data to dictate the analytic categories [26]. After coding each transcript using coding categories that were mutually agreed upon by three study team members, contents of each coding category were reviewed to ensure agreement on the nature of respondents’ responses to the interview questions. Three steps were taken to increase methodological rigor: 1) multiple investigators participated in data collection and analysis to ensure multiple viewpoints and discussion of perceptions of data, 2) three investigators identified emerging codes through weekly reviews to refine the coding scheme, ensure consensus, and establish consistency across coders, and 3) 20% of the interviews were double-coded for inter-coder reliability and coders agreed on 84% of the coding. The remaining coding inconsistencies were resolved by group discussion and re-coded for the final dataset.

## Results

Table 2 summarizes the five challenges to participant recruitment and enrollment that emerged in the qualitative analysis: eligibility criteria, stigma, research related complexities, patient preferences, and social and structural barriers. Specific barriers were identified within each challenge and sample quotations describe the barriers.

**Table 2:** Barriers inhibiting study recruitment and sample quotation

Theme	Specific barrier	Quote	Potential Actionable steps
1. Eligibility Criteria	Suppressed HIV viral loads	People that are showing up to an HIV clinic even sporadically have a very high suppression rate. 85 to 90%, because the medicines have just gotten so much easier. It really is hard to find these folks if you are sitting in the clinic.	Consider broadening eligibility criteria to include individuals with unsuppressed HIV or drugs of choice in a specific community. Peer outreach workers or partnering with other organizations can be helpful recruitment opportunities.
	Opioids not the primary drug	There’s not as many opioid users at least here as in other parts [of the country]. Among [our] population, [there is] more methamphetamine use.	
2. Stigma	Fear of learning HIV status	The stories of people’s fears when we talk about their diagnosis experiences and we talk about their reactions and disclosures and all of that it’s like we’re back in the 80s, early 90s. Especially in [our rural community].	Ensure that staff are well trained on the stigma that patients feel and can respond in manner that makes them feel comfortable in the clinic.
	Fear of others learning their HIV status	Because if you live in a town of a couple thousand people, it’s very ‘somebody that knows somebody that knows somebody’ sees you walking into this [HIV] clinic. This is why we have people driving several hours one way to come here. It gets incredibly difficult to even locate individuals who may be susceptible, who may be in need of MAT	
	Internalized stigma and self-shaming	It’s kind of this self-shaming thing like ‘I did it to myself, I deserve to have [HIV].’ There’s a lot of cultural stigma and shame surrounding HIV and Hepatitis C.…People often report their substance use and do not tell me that they tested positive for Hep C or HIV.	
3. Research complexities	Lengthy procedures	The [patients] are [thinking], ‘I’m sitting here for three hours, I could be out on the street making money to get well.’ At this point a lot of them don’t even enjoy the high, but they have to keep using to not get sick.	Ensure that the research procedures are as streamlined as possible while providing adequate time to answer all questions and concerns.
	Fear of research and outsiders	The older generation especially the older black men. Definitely. They are like … remember what happened … when they gave all those black men syphilis? How will I know you are not doing that?	
4. Patient preferences	Treatment preference	I’ve had people who were randomized into treatment as usual [say] ‘I was looking forward to getting the injection’ and vice versa.	Provide all of the information to the patient about the pros and cons of each medication. Make sure they are comfortable with either study condition prior to randomization so that study resources are used for patients who are willing to follow through for either arm.
		A lot of patients still view [buprenorphine] and methadone as opioids ... Once they understand what [XR-NTX] is, they don’t see that as a quote dependent drug. Some people actually preferentially desire to get onto [XR-NTX] …	
	Concerns about withdrawal	When I describe precipitated withdrawal, people say, ‘Oh, is that like what happens after I use [naloxone]?’ If they have ever done that they are petrified of using [XR-NTX] because they never want to feel like that again.	
5. Social and structural barriers	Housing and transportation	[Patient] had housing issues and that seemed to take priority before being able to stop using because she couldn’t go to inpatient just yet because she didn’t feel secure with her housing situation.	The extent possible, assist patients with wrap-around services such as housing referrals or transportation services. Understand any criminal justice involvement and how to track them should they become incarcerated.
		Mass transit in [our community] is not great. Buses run late, there’s lots of traffic. … you have to make so many connections and when you combine the amount of time and the amount [of] delays, it’s very hard for people to make appointments on time.	
	Criminal justice involvement	You could be sitting with them doing an assessment and then the next minute they walk out of your office and boom, they are arrested, you know, you-- it’s a revolving door.	

### Eligibility criteria

Study eligibility criteria required co-occurring OUD and HIV viremia (i.e., unsuppressed viral load); respondents reported difficulty locating patients with unsuppressed HIV viral load and OUD within the HIV clinic and, in some communities illicit drugs other than opioids were more common among HIV patients.

#### Suppressed HIV viral loads

The requirement for a participant to have an HIV RNA load of more than 200 copies/mL was particularly challenging because viral suppression is increasingly easier to achieve among PLWH who are engaged in HIV treatment [27].

Our viral suppression rate for our HIV patients is about 81, or 82 percent so, I mean, we have a high viral suppression rate.

Few patients already receiving care at HIV clinic study sites, therefore, were eligible to participate in the study. In order to locate PWLH with unsuppressed viral loads, the research team had to identify and screen individuals less engaged with care, often through HIV testing of at-risk individuals.

For us it's a gigantic learning curve to figure out how to reach out into the community because we've really depended on patients walking through our door. [For this study,] we can't wait for patients to come in.

People that are showing up to an HIV clinic even sporadically have a very high suppression rate. You know, 85, 90 percent, because the medicines have just gotten so much easier. It really is hard to find these folks if you are sitting in the clinic.

#### Opioids not primary drug in the community

Some sites found recruitment challenging because opioids were not the primary drug used in the community. One site had a database of individuals that could help them find the target population. Because methamphetamine was the primary drug in the community, however, the site struggled to find PLWH who met all the criteria for inclusion.

There's not as many opioid users at least here as in other parts. Among this population, [there is] more methamphetamine use. I think while it's looked at nationally, there are regional differences.

Opioids have been in the national spotlight, but community differences in the epidemic are easily obscured. As the inclusion/exclusion criteria become more specific, it is important to consider the impact of each criterion on recruitment within a specific community.

### Stigma

HIV and SUD related stigmas were potent patient barriers to study recruitment. Themes emerged related to internalized stigma and prospective study participants’ fears of learning their HIV status. Other challenges included repercussions associated with their communities learning of their HIV status.

#### Fear of learning HIV status

Respondents frequently mentioned how prospective participants’ fears of learning their HIV status interfered with study recruitment: “I think we’ve learned through the study that there’s a huge barrier just to get tested for HIV.” Patients conveyed outdated notions of what an HIV diagnosis means despite the great medical advancements in HIV treatment: “We’ve had patients that upon hearing the diagnosis of HIV think that they’re going to die the next day, that it’s a death sentence immediately.” At a rural site, a respondent explained:

The stories of people’s fears when we talk about their diagnosis experiences and we talk about their reactions and disclosures and all of that it’s like we’re back in the 80s, early 90s. Especially in [our rural community]. People feel like they have to bleach their bodies, because they’re dirty.

One site distributed harm reduction supplies (e.g., sterile syringes, sharps containers for safe disposal, wound care supplies and fentanyl test strips) to encourage study participation. Study staff discussed the study with prospective participants, developed rapport with the target population, and attempted to allay their concerns about learning their HIV status.

#### Fear of others learning of their HIV status

Respondents reported that stigma around HIV was a more potent barrier to recruitment than having others learn of their SUD. Respondents reported that, coupled with the fear of learning their own HIV status, prospective study participants were concerned about family and community members discovering their HIV status. Because if you live in a town of a couple thousand people, it’s very ‘somebody that knows somebody that knows somebody’ sees you walking into this [HIV] clinic. This is why we have people driving several hours one way to come here. It gets incredibly difficult to even locate individuals who may be susceptible, who may be in need of MAT (medications for addiction treatment), much less get them in and get them initiated [into the study].”

The implications varied, with some very personal consequences. One respondent explained that “It’s common practice [in] our rural HIV positive people that, if they’ve disclosed to their family, they must eat off of disposable plates and silverware.” While this example is certainly stigmatizing and uncomfortable, other repercussions can be much more severe. A clinician reported that a patient had disclosed that members in their community “… might kill them if they learned they were HIV positive.” As a result of these patient concerns, study follow up and communication were impacted:

I can’t tell you how often in the clinic I hear someone saying ‘Well you can’t send any mail to my house that might come from the clinic, and you can’t send my medicines to that pharmacy because the guy who works at the pharmacy knows my cousin and the whole county will be talking.’ Everybody’s up in everybody’s business. It even affects how we communicate results.

Because there is less bias against SUD, one solution was to use recruitment strategies that focus on the OUD eligibility criterion before HIV status:

It's very hard for the people in our community to walk in our door thinking that someone is going to find out that they have HIV. That's why when we are recruiting people we are … using the substance use problem as the way to get people in. We will screen them when they are here if they have HIV.

#### Internalized stigma and self-shaming

Some respondents reported that when talking with prospective participants, the participant disclosed feeling ashamed and isolated, as if “there’s no one else like me.” These feelings of internalized stigma and shame interfered with completing an accurate patient history.:

It's kind of this self-shaming thing like ‘I did it to myself, I deserve to have this’. There's a lot of cultural stigma and shame surrounding HIV and Hepatitis C. I've found less reported shame surrounding substance misuse disorders. People often report their substance use and do not tell me that they either tested positive for Hep C or HIV. Or if I ask the question, people tend to shut down and that takes longer for me to get that history than the substance use history.

Respondents sough to overcome these uncomfortable feelings with a welcoming and safe environment.

To overcome stigma and build trust while recruiting in the community, study sites used peer outreach workers to extend recruitment outside of the HIV clinic and reach people actively injecting drugs and living on the streets. One of the more successful recruitment sites had an experienced full-time outreach worker that built rapport by distributing safer sex supplies in the community: “I also pass out condoms, [and] be friendly with the drug dealers just to let them know that I’m no threat.”

### Research complexities

Study enrollment required substantial time to complete necessary blood draws, a detailed consent form, a detailed psychosocial history and confirm an opioid use disorder diagnosis. Some respondents reported that prospective study participants had a fear of research, researchers, and “outsiders” in general which inhibited willingness to participate in the study.

#### Lengthy enrollment procedures

Some potential participants had difficulty sitting through all the recruitment paperwork when they became anxious and fidgety due to opioid withdrawal symptoms. As a result, the recruitment process could take several days. Attempts to break up one comprehensive enrollment visit into several briefer, more tolerable visits were not helpful. This approach increased the risk that sites would lose participants or need to begin the screening process again if they did not return on time for subsequent visits.

It takes less time if we do it all at once. … [I could say] ‘We can do this part on one day, and you can come back a few days later and we can do this part. Then it’s less time for each visit.’ With this population, you can’t do that. If you say, ‘Hey come in for this part today and then in a couple days when you have more time you can come in and do this next part,’ they’re just gone.

One site reported that the enrollment process was smoother for participants who were recruited during hospitalization. This was facilitated in part by lab tests (i.e. HIV antibody and RNA tests) which had already been drawn during the hospital admission. Clinics affiliated with hospitals or other clinics developed or utilized internal referral systems to identify out-of-care patients and patients that had been hospitalized:

That’s one of the better recruitment methods that we have going for us right now is our internal referrals. If it’s a person who has fallen out of care, they’ll send a message that says ‘hey, so-and-so is in the hospital’.

#### Fear of Research and outsiders

During outreach activities, study staff encountered skepticism of research, researchers, and more generally, outsiders coming into their community for recruitment activities. Within the African American community, some prospective study participants were reluctant to engage with study staff due to the history of unethical research conduct in their communities [28]. A research assistant explained:

I know the people that I talk to; they always feel like black people are being targeted … The older generation yeah, they all complain about research studies. Especially the older black men. Definitely. They are like -- I guess they don't really know their names-- the Tuskegee study -- but they are like remember what happened with all those black men when they gave all those black men syphilis? How will I know you are not doing that?”

Another respondent echoed these experiences, noting that potential participants ask “Well what about Tuskegee? People are experimenting on us.” Staff worked to overcome this barrier by slowly developing trust and rapport and by communicating how potential subjects will be protected.

I just say ‘I appreciate you respecting me and trusting me enough to listen and learn more about the study to just make a better decision’ … They are starting to be more open.

Concerns about research were not limited to African American communities. At the rural site serving a low-income white community, concerns about research participation emerged:

There’s skepticism about the university in [our] community; it’s like a research institution so they’re already a little bit on guard when you’re trying to do this kind of research. It’s just huge.

Another respondent working at a site in the Southern U.S. noted that her status as an outsider to the rural community posed a barrier to recruitment: “And even without the color issue and the race issue, even myself as a Caucasian female, I’m Mid-western and I may or may not be accepted”. Another solution for gaining entrée to the target population has been working with syringe exchange programs to get referrals.

The needle exchange has been a god send. People come to them to exchange needles and … they have HIV testing. Once an HIV positive individual is identified, they immediately contact us.

### Patient preferences

During recruitment, study staff discussed with participants the importance of being open to either treatment arm and sought individuals with “willingness and readiness and commitment”. Some prospective participants had a preference for either opioid agonist therapy (TAU) or opioid antagonist therapy (XR-NTX).

I've had people who were randomized into TAU and were like ‘I was looking forward to getting the injection’ and then vice versa. We've had some TAU's that are like ‘Okay, you know, this is an opportunity, let me take advantage of it’ and the same goes for [XR-NTX].

When respondents were asked how individuals formed these opinions and preferences, a research assistant explained, “I think a lot of it is community-based knowledge,” and that “word of mouth can have tremendous impact.” In one case, a patient randomized to XR-NTX did not have a positive experience and shared this liberally within her community. The research associate commented that this early participant had a negative impact on subsequent recruitment as “that word spread and made other people leery”.

Other concerns that seemed to prevent prospective participants from engaging in the study included fear of needles (used for the XR-NTX injection) and negative associations with XR-NTX because of its use within jails and drug courts: “There’s this negative connotation because for a lot of our patients [XR-NTX] equals the criminal justice system.”

Because naltrexone is an opioid antagonist, pain management was also a concern with XR-NTX:

I’ve had a couple of patients who were like “What if I need a dental procedure?” or “What if I have to have surgery and I’m on this medicine?” So that’s going around too.

Study staff have worked to overcome these concerns by discussing non-opioid pain management alternatives.

#### Substituting a “drug for a drug”

Some prospective study participants did not perceive the use of opioid agonist therapy as being drug-free. A research assistant explained that they often heard comments such as “Oh, if I’m going to kick this, it’s going to be on my own, and through God. It’s not going to be that I’m addicted to another substance – to a legal heroin”. At a clinic with rural patients, the 12-step programs in the community were described by staff as “very much abstinence based”, and held that agonist therapies are “trading a drug for a drug”. In these areas, XR-NTX had an advantage for recruiting prospective participants.

A lot of patients still view [buprenorphine] and methadone as opioids and depending on something. Once they understand what [XR-NTX] is, they don't see that as a quote dependent drug. Some people actually preferentially desire to get onto [XR-NTX] … and, despite all odds, manage to get on it.

*Concerns about withdrawal.* Participants randomized to the XR-NTX arm must be opioid free prior to induction to prevent precipitated withdrawal. Patients’ concerns about opioid withdrawal symptoms were recruitment barriers: A lot of our OUD clients have an intolerance of distress and pain and feeling uncomfortable. They’re just not ready to make that leap.

As part of the induction protocol, clinicians may administer a naloxone challenge to confirm that patients are opioid-free prior to the first XR-NTX injection. Patients who had prior experiences receiving naloxone to reverse an overdose were apprehensive about the possibility of precipitated withdrawal effects:

When I describe precipitated withdrawal, people then will say, ‘Oh, is that like what happens after I use [naloxone]?’ If they have ever done that they are petrified of using [XR-NTX] because they never want to feel like that again.

A facilitating factor, conversely, for recruitment was the familiarity of prospective participants with buprenorphine:

Most of the patients that we have, when they come to us they’re super interested in [buprenorphine] because … there is a black market and illicit street use for [buprenorphine]. A lot of people are treating themselves. They see their community dying from heroin and from fentanyl overdoses and they get scared. They buy [buprenorphine] off the street from their friend and treat themselves … People … have experience with buprenorphine. They know it works. They know they feel normal on it. They know they don’t have withdrawal if they do it correctly.

### Social and structural barriers

Many prospective study participants faced challenging social and structural barriers including homelessness or living environments with elevated rates of substance use, criminal justice involvement, and lack of transportation. These factors, along with ambivalence about treatment, could impact their willingness to engage in the study.

#### Housing, communication, and transportation

Homelessness, phone problems, and unreliable transportation impeded prospective participants’ ability to engage in the study. Many potential participants did not have phones, changed phones frequently, or “do not have minutes on their phone” to maintain contact with research staff. Communication with staff was crucial, as it may take several days for the patients to complete the screening process, and they must return for scheduled appointments:

There is no routine to their life so sometimes you know, weekends run into weekdays and they might not really remember that they have an appointment There's no way to contact them to remind them, you know? Something as basic as that; they just don't have a way to remember.

Turbulent living conditions were considered to be a universal barrier to recruitment: “Home is not a safe place for a lot of people and so asking someone to be there when there's all of the same kind of stressors and inducement is really challenging”. For some, addressing chaotic home situations takes precedence over treatment. [Patient] had housing issues and that seemed to take priority before being able to stop using because she couldn't go to inpatient just yet because she didn't feel secure with her housing situation.

A clinician explained that in her clinic, recruiters “might have hooked in with somebody but then the housing falls through and then we lose them”.

Inadequate transportation was also a common barrier. For some, this was because of the large service area. A respondent explained that their clinic serves more than 60 counties and “Some of the people drive over two hours one way just to come to their clinic visits, so it can be difficult to motivate even the ideal participant to come to research visits.” Sites that recruited primarily from urban settings also reported that transportation was a barrier due to inconsistent public transit and heavy traffic:

Mass transit in [our community] is not great. Buses run late, there's lots of traffic. It takes a long time to get from point A to point B because you have to make so many connections and when you combine the amount of time and the amount [of] delays, it's very hard for people to make appointments on time.

Access to a clinic vehicle was helpful for addressing the transportation barrier by picking up and dropping off participants for their enrollment visits. However, this was only an option for those sites that already had this option in place. This was primarily because the site had already addressed issues such as safety, privacy, liability, and insurance.

We transport them ourselves in this van. I can't imagine having to set that up at the beginning of this trial. It's something that took a lot of time and effort to figure out and thankfully it worked well.

Some sites also utilized rideshares such as Uber and Lyft to address transportation barriers.

#### Legal system involvement

Prospective participants’ on-going participation in illicit activities such as purchasing drugs, sex work or “hustling for money” brought them into contact with the legal system on a regular basis. In some cases, this contact interrupted the enrollment process.

It's a revolving door with our clients. You could be sitting with them doing an assessment and then the next minute they walk out of your office and boom, they are arrested, you know, you-- it's a revolving door.

In one example, a participant had successfully completed all of the screening requirements and had been randomized to the XR-NTX arm of the study. Nonetheless, induction was disrupted.

We just tried so hard to get her onto the [XR-NTX] shot and she just wasn't ready because of a lot of different things going on in her life and then she was incarcerated.

#### Readiness to change

Coupled with social and structural difficulties, prospective study participants also faced challenges related to “readiness-to-change” and begin substance abuse treatment:

A lot of people are scared to stop like-- if I'm sober, if I don't use drugs anymore, then what? So if they can have that support like, this is what we are going to do like the hand-holding stuff-- we are going to do this now.

Several respondents noted patients’ reluctance to engage, even after the extensive screening process was completed.

When they are randomized into whatever group that they are put into, that's when they realize ‘uh oh, no, I don't want to do this’ or ‘I'm not ready’. During the pre-screening, screening, randomization all that section there, they are fine, they are great initially and then when they are faced with-- you've been put in the [XR-NTX] section or the TAU, it's like they start to shuffle. I guess the not readiness or the not decisiveness or their willingness to change

A factor that has motivated readiness for treatment in some patients has been the sharp increase in overdose deaths in their communities. The adulteration of heroin with fentanyl has had a chilling effect on those who are seeing their friends and loved ones die.

People are dying. People who have successfully been using and doing relatively well, not over-dosing, still alive for thirty, forty years, now are having friends that are dying and have overdosed. Coming and saying I'm scared that I could die, the stuff that's out there is not what it used to be. I got to do something about it.

Recruiters used motivational interviewing techniques to assist patients who were feeling ready “to do something” but perhaps still ambivalent. Motivational interviews helped prospective patients see the costs and risks of continued use.

The motivational interviewing is helpful in getting to that point, in teasing out the information that we would need in order to address any concerns the participant might have in order to help them recognize what their wants are.

These “wants” and goals of participants varied and included desires to “rekindle relationships, make things right”, re-gain custody of children, find employment and financial stability, establish stable housing and other factors.

## Conclusion

Respondents reported barriers that hindered recruitment and enrollment and methods to overcome these barriers to enrollment in the CTN-0067 CHOICES clinical trial. As these barriers came into focus, study sites found strategies to facilitate enrollment. For example, patient transportation barriers could be overcome by use of a clinic vehicle. Rideshares are a viable alternative solution but required additional funding.

Patient attitudes and preferences for a particular study drug affected study participation. While site staff worked to ensure that potential participants were willing to be assigned to either XR-NTX or buprenorphine, staff also reported that participants retained preferences despite agreeing to be assigned to either medication. This resulted in some participants being lost to follow up when they were not randomized to the medication they preferred. Treatment preference was associated with study drug initiation and retention in a recent comparative effectiveness trial of XR-NTX versus buprenorphine/naloxone in patients admitted for medically supervised withdrawal [23].

Study sites expanded recruitment strategies to include individuals who were new to HIV treatment and people with opioid use disorders who were either out-of-HIV treatment or not previously diagnosed with an HIV infection. The most successful sites a) developed local partnerships with other organizations or referral sources; b) had strong street-level outreach activities and the use of peer patient navigators; and c) forged systems for internal referrals when patients were hospitalized.

Several limitations of the study should be acknowledged. The study was exploratory and assessed barriers to recruitment and enrollment in a national multisite randomized trial of XR-NTX vs. TAU for the treatment of OUD in persons with unsuppressed HIV disease. Thus, the findings from this study may not generalize to other trials of substance use disorder treatment or those involving persons without HIV infection. The study focused on provider perspectives; future studies will examine the barriers and facilitators to enrollment from the patient perspective. The small sample size early in enrollment did not allow for statistical tests to examine differences by organizational or individual demographics such as recruitment site or participant race. Quantitative analysis of associations between individual demographics, organizational characteristic and study recruitment and retention will be assessed when enrollment targets are met.

Persons with untreated HIV and OUD can be enrolled in multisite clinical trials by using enhanced recruitment strategies that extend outside of the HIV clinic. Employing peer outreach workers and collaborating with syringe service programs may be especially helpful in facilitating recruitment and merit inclusion in the study protocol and its implementation. Respondents’ perspectives provide valuable information for designing, implementing, and evaluating recruitment and enrollment strategies for other substance use disorder treatment studies and services for persons living with HIV. Results demonstrate the importance of these barriers for SUD researchers and, by extension, treatment providers.

This exploratory analysis examined barriers and facilitators to study recruitment and enrollment in the CTN-0067 CHOICES clinical trial comparing the effectiveness of XR-NTX versus TAU for HIV-infected persons with opioid use disorder. Respondents identified a range of issues that impeded patient participation in the study including stigma, narrow eligibility criteria, lengthy enrollment procedures, patient preferences about study arm, and social and structural factors that increase patient complexity. Although it was not possible to alter our eligibility criteria, future studies may want to consider broadening eligibility criteria to include individuals with unsuppressed HIV or targeting the varying drugs of choice in a specific community. Helpful tactics study sites were able to employ included ensuring that staff understood the stigma that patients feel and making them feel as comfortable as possible in the clinic. This included trying to ensure that the research procedures were as streamlined as possible while providing adequate time to answer all questions and concerns. Staff also worked diligently to provide all of the information to the patients about the pros and cons of each medication, so that they were willing to enroll in the study despite the condition they were randomized to. Though study personnel encountered barriers to recruitment in the CHOICES study, persons with untreated HIV and OUD can be enrolled in multisite clinical trials by using enhanced recruitment strategies that extend outside of the HIV clinic. Employing peer outreach workers and collaborating with syringe service programs may be especially helpful in facilitating recruitment and merit inclusion in similar study protocols.

## Abbreviations

HIV: Human immunodeficiency virus
OUD: Opioid use disorder
PLWH: People Living with HIV
TAU: treatment as usual
XR-NTX: Extended Release Naltrexone
